# Association between abdominal muscle mass quantified by computed tomography and depression in middle-aged and older Korean men: a cross-sectional study of 2,877 cases

**DOI:** 10.3389/fmed.2026.1656330

**Published:** 2026-03-12

**Authors:** Gyeonghwa Jeong, Young-Jee Jeon, Doo-Ho Lim

**Affiliations:** 1 Department of Internal Medicine, Ulsan University Hospital, University of Ulsan College of Medicine, Ulsan, Republic of Korea; 2 Department of Family Medicine, Ulsan University Hospital, University of Ulsan College of Medicine, Ulsan, Republic of Korea

**Keywords:** abdominal muscle mass, computed tomography, depression, older men, sarcopenia

## Abstract

**Background:**

Depression is a prevalent mental health concern in aging populations and is associated with increased morbidity and reduced quality of life. While psychosocial and biochemical factors have been widely studied, the role of muscle mass, particularly abdominal muscle mass, in depression remains unclear. This study aimed to investigate the association between abdominal muscle mass, as measured by computed tomography (CT), and depression in middle-aged and older Korean men.

**Methods:**

This retrospective cross-sectional study included 2,877 men aged ≥ 40 years who underwent both abdominopelvic CT and the Beck Depression Inventory (BDI) as part of a general health examination. Abdominal muscle mass was quantified using the total abdominal muscle area (TAMA) at the L3 vertebral level. Depression was defined as a BDI score ≥ 16. Multivariable logistic regression analyses were performed to examine the association between TAMA and depression, adjusting for age, body mass index, lifestyle factors, comorbidities, and biochemical markers.

**Results:**

The prevalence of depression decreased across increasing TAMA quartiles (*p* = 0.018). Higher TAMA was independently associated with a lower risk of depression. In the fully adjusted model, participants in the highest TAMA quartile had a significantly reduced odds of depression compared to those in the lowest quartile (adjusted OR, 0.534; 95% CI, 0.297–0.958; *p* = 0.035).

**Conclusion:**

Greater abdominal muscle mass, as assessed by CT, was significantly associated with a lower prevalence of depression in middle-aged and older Korean men. These findings suggest that abdominal muscle mass may be a marker of mental health during aging.

## Introduction

Depression is a prevalent mental health disorder impacting millions globally, with a notable incidence in aging populations (1). Among older adults, depression frequently remains underdiagnosed and inadequately treated, despite its association with substantial functional decline, diminished quality of life, and elevated mortality rates (2, 3). Identifying potential risk or protective factors for depression within this population is a critical public health priority.

Skeletal muscle mass has recently gained attention as a possible correlate of mental health. Several studies have reported that sarcopenia, or age-related loss of muscle mass and strength, may be associated with an increased risk of depressive symptoms (4). However, most prior research has focused on appendicular muscle mass or whole-body composition, often assessed by handgrip strength, dual-energy X-ray absorptiometry (DXA), or bioelectrical impedance analysis (BIA) (5–7). The relationship between regional muscle mass, particularly abdominal muscle mass, and depression remains incompletely understood.

Abdominal muscle mass, which includes core and postural muscles, plays a key role in physical function and metabolic health (8, 9). Computed tomography (CT) provides a highly precise and reproducible technique for quantifying abdominal muscle mass and distinguishing between muscle and fat compartments (10). As CT imaging becomes more prevalent in clinical practice, it presents a valuable opportunity to investigate novel imaging biomarkers pertinent to both physical and mental health. Despite its potential significance, limited research has examined the relationship between CT-measured abdominal muscle mass and depression in aging populations. This study concentrated on men due to sex differences in body composition, muscle distribution, and depression risk, which may affect this relationship (11, 12), and sex-specific analyses can yield more targeted insights.

Therefore, the aim of this study was to investigate the association between abdominal muscle mass, as quantified by CT, and the prevalence of depression in middle-aged and older Korean men.

## Materials and methods

### Study design and participants

**FIGURE 1:**
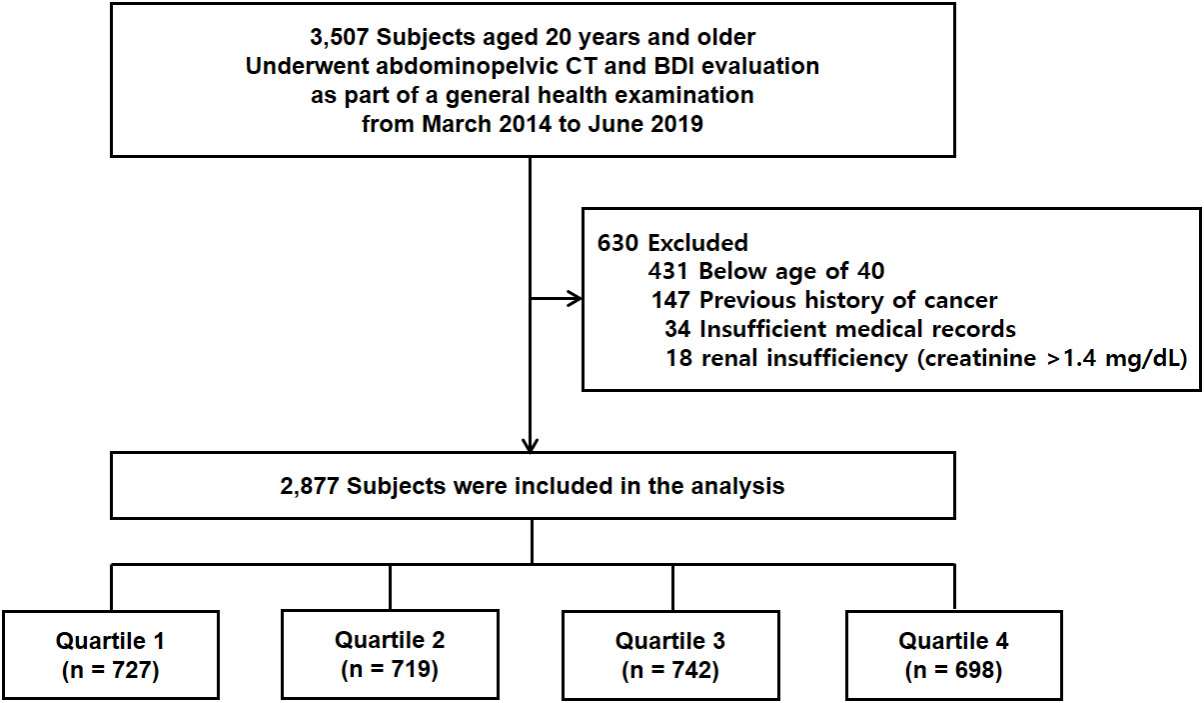
Overview of the study population. CT, computed tomography; BDI, Beck Depression Inventory.

This retrospective cross-sectional study included 3,507 participants who underwent both abdominopelvic CT and the Beck Depression Inventory (BDI) as part of a general health examination at a single medical center between March 2014 and June 2019. Participants were excluded if they met any of the following criteria: age < 40 years (*n* = 431), a prior history of cancer (*n* = 147), incomplete or missing clinical data (*n* = 34), or renal insufficiency (*n* = 18). After applying these exclusion criteria, a total of 2,877 individuals were included in the final analysis (Figure 1).

### Clinical and laboratory measurements

Clinical and laboratory data were extracted from electronic medical records and an institutional clinical data warehouse platform. Measurements of height, body weight, and blood pressure were conducted in accordance with standard protocols during the general health examination (13). Venous blood samples were collected following an overnight fast and analyzed using standard laboratory procedures. Hematologic assessments included white blood cell count, hemoglobin concentration, and platelet count. Biochemical parameters evaluated comprised serum total bilirubin, albumin, aspartate aminotransferase (AST), alanine aminotransferase (ALT), total cholesterol, fasting blood glucose, and hemoglobin A1c. Diabetes was defined as fasting plasma glucose ≥ 126 mg/dL, hemoglobin A1c ≥ 6.5%, or a self-reported history of diabetes or treatment with dietary modifications or antidiabetic medications. Hypertension was defined as a systolic blood pressure ≥ 140 mmHg, diastolic blood pressure ≥ 90 mmHg, or a self-reported history of hypertension or treatment with antihypertensive medication (13).

### Definition of physical activity

A survey was conducted to assess the physical activity levels of participants in accordance with the World Health Organization’s Global Recommendations on Physical Activity for Health 2010 (14). Participants were classified as physically active if they engaged in a minimum of 150 min of moderate-intensity aerobic exercise or at least 75 min of vigorous-intensity exercise per week, with each aerobic session lasting no less than 10 min.

### Definition of alcohol use

Alcohol intake was evaluated via a self-administered questionnaire completed during routine health examinations. Individuals who reported consuming alcoholic beverages on one or more occasions per week were categorized as alcohol users, whereas participants reporting drinking less frequently or abstaining altogether were categorized as non-users (15).

### Measurement of abdominal muscle mass

The assessment of abdominal muscle mass was conducted using CT. Image acquisition was performed with a SOMATOM Definition Flash scanner (Siemens Healthcare, Erlangen, Germany). For contrast enhancement, 100–120 mL of iopromide (Xenetix 350; Guerbet, Roissy, France, 150 mg/mL) was administered intravenously through an 18-gauge cubital vein at a rate of 3–4 mL/s, followed by a 20 mL saline flush. Enhanced images were obtained 80 s post-contrast administration. The CT protocol included a beam collimation of 128 mm × 0.6 mm, a pitch of 0.6, a gantry rotation time of 0.5 s, and a field of view (FOV) adjusted to patient size at 100 kVp. Radiation dose optimization was achieved using the CARE Dose 4D system (Siemens Medical Solutions, Erlangen, Germany). Images were reconstructed using an I40f kernel with a slice thickness of 3 mm (16). The reproducibility of the TAMA measurement was excellent, with an intra-rate intraclass coefficient (ICC) of 0.97 and an inter-rater ICC of 0.95.

Body composition analysis was performed on abdominopelvic CT images using the Asan-J software, a customized program based on ImageJ (National Institutes of Health, Bethesda, MD, USA) (17). Two consecutive axial images at the level of the inferior endplate of the third lumbar vertebra (L3) were selected and averaged for each subject. The total abdominal muscle area (TAMA), including the psoas, paraspinal, transversus abdominis, rectus abdominis, quadratus lumborum, and internal and external oblique muscles, was quantified in cm^2^ based on predefined Hounsfield unit (HU) thresholds (−29 to +150 HU). TAMA was further categorized into low-attenuation abdominal muscle area (LAMA) and normal-attenuation abdominal muscle area (NAMA) based on CT HUs (TAMA, −29 to 150 HU; LAMA, −29 to 29 HU; NAMA, 30–150 HU). The extramyocellular lipid area (EMCLA), indicative of lipid infiltration within the muscle compartment, was defined using thresholds between −190 and −30 HU. Intermuscular fat was excluded from all muscle area (Supplementary Figure 1) (16).

### Evaluation of depression

The Beck Depression Inventory (BDI), a self-report questionnaire, was developed to evaluate the severity of depressive symptoms. This inventory comprises 21 items, each rated on a 4-point Likert scale. The response scores for each item range from 0 to 3, resulting in a total score range of 0–63, with higher scores indicating more severe depressive symptoms (18). The BDI demonstrates high internal consistency, with Cronbach’s alpha coefficients of 0.86 and 0.81 for psychiatric and non-psychiatric populations, respectively. The Korean version of the BDI has been validated in a prior study, which suggested 16 points as the optimal cutoff value for diagnosing depression (19).

### Statistical analysis

Participants were stratified into quartiles according to TAMA. Categorical variables were represented as frequencies and percentages, whereas continuous variables were summarized as means ± standard deviations. Group comparisons were conducted using Pearson’s chi-square test or Fisher’s exact test for categorical variables, and one-way analysis of variance (ANOVA) or the Kruskal–Wallis test for continuous variables, as appropriate. To investigate the association between TAMA and the prevalence of depression, multivariable logistic regression analyses were performed. Covariates for adjustment were selected based on clinical relevance and statistical significance. Four models were constructed in a stepwise manner: the initial model (Model 1) included no adjustments. Model 2 was adjusted for age and body mass index (BMI). Model 3 expanded on this by incorporating lifestyle and clinical factors, such as smoking status, alcohol use status, physical activity status, hypertension, and diabetes. Model 4 further included biochemical markers such as hemoglobin, albumin, total bilirubin, AST, ALT, total cholesterol, and hemoglobin A1c. Multicollinearity between TAMA and BMI was assessed using variance inflation factor (VIF). The VIF value for BMI was 1.687, indicating no significant multicollinearity (VIF < 5) (Supplementary Table 1). Model calibration was evaluated using the Hosmer–Lemeshow test and calibration plots, while discrimination was assessed by the area under the receiver operating characteristic curve (AUC). Pseudo-R^2^ values were computed to evaluate model fit. The linearity of the logit was examined using the Box–Tidwell procedure (Supplementary Table 2). Missing data was <2% for all covariates; therefore, complete case analysis was performed. Given the overall number of depression events (>170 cases) relative to the number of covariates, the events-per-variable ratio exceeded commonly accepted thresholds (≥10), indicating a low risk of overfitting. Results from the logistic regression analyses are presented as adjusted odds ratios (ORs) with 95% confidence intervals (CIs). All statistical tests were two-tailed, and a *p*-value < 0.05 was considered statistically significant. Data management and statistical analyses were conducted using SPSS software (version 24.0; SPSS Inc., Chicago, IL, USA).

### Ethical statement

This study adhered to the ethical guidelines of the Declaration of Helsinki (revised in Brazil in 2013) and was approved by the Institutional Review Board of UUH (IRB No. UUH 2024-12-028).

## Results

The mean age of the 2,877 participants was 55.0 ± 7.6 years, with a mean TAMA of 156.1 ± 22.5 cm^2^. Table 1 presents the baseline characteristics of the study population stratified by TAMA quartiles. Participants in the highest TAMA quartile were significantly younger and exhibited a higher BMI compared to those in the lowest quartile. The prevalence of diabetes mellitus decreased across the quartiles, whereas no significant trend was observed for hypertension. The proportion of alcohol users increased significantly across the quartiles. Several laboratory parameters, including hemoglobin, albumin, total bilirubin, AST, ALT, and total cholesterol, demonstrated significant increases with higher TAMA levels. No significant differences were observed across quartiles for platelet count, fasting glucose, or hemoglobin A1c.

**TABLE 1:** Baseline characteristics of the study population according to the quartiles of total abdominal muscle area.

Characteristics	Overall (*n* = 2,877)	Total abdominal muscle area				
		Quartile 1 ≤140.0 cm^2^ (*n* = 727)	Quartile 2 140.1 ∼ 155.0 cm^2^ (*n* = 719)	Quartile 3 155.1 ∼ 171.0 cm^2^ (*n* = 742)	Quartile 4 ≥171.1 cm^2^ (*n* = 698)	*P*-value
Age, years	55.0 ± 7.6	58.4 ± 7.9	55.6 ± 7.3	54.2 ± 7.1	51.5 ± 6.3	<0.001
BMI, kg/m^2^	24.4 ± 2.8	22.2 ± 2.2	24.0 ± 2.1	25.1 ± 2.2	26.7 ± 2.4	<0.001
Diabetes mellitus	331 (11.5)	109 (15.0)	78 (10.8)	86 (11.6)	58 (8.4)	0.001
Hypertension	730 (25.4)	180 (24.8)	177 (24.6)	204 (27.5)	169 (24.5)	0.497
Current smoker	1036 (36.0)	263 (36.2)	227 (31.6)	264 (35.6)	282 (40.9)	0.004
Alcohol user	2270 (78.9)	533 (73.3)	556 (77.3)	586 (79.0)	595 (86.4)	<0.001
Physically active	579 (20.1)	132 (18.2)	148 (20.6)	154 (20.8)	145 (21.0)	0.495
White blood cell, K/mmL	5.793 ± 1.7	5.878 ± 1.8	5.766 ± 1.7	5.729 ± 1.7	5.802 ± 1.6	0.383
Hemoglobin, g/dL	15.2 ± 1.2	14.9 ± 1.3	15.2 ± 1.1	15.2 ± 1.2	15.5 ± 1.1	<0.001
Platelet, K/mmL	223.6 ± 49.5	224.9 ± 52.4	224.2 ± 48.9	222.7 ± 49.6	222.4 ± 47.1	0.750
Albumin, g/dL	4.51 ± 0.38	4.48 ± 0.40	4.51 ± 0.37	4.50 ± 0.39	4.55 ± 0.35	0.006
Total bilirubin, mg/dL	0.99 ± 0.43	0.92 ± 0.36	0.99 ± 0.44	1.01 ± 0.43	1.05 ± 0.46	<0.001
AST, IU/L	27.7 ± 15.4	27.4 ± 16.8	26.9 ± 13.5	27.2 ± 12.0	29.5 ± 18.5	0.006
ALT, IU/L	31.6 ± 20.0	28.9 ± 17.6	30.8 ± 18.8	31.4 ± 18.5	35.5 ± 24.2	<0.001
Total cholesterol, mg/dL	185.4 ± 39.0	182.9 ± 41.3	185.3 ± 38.8	182.9 ± 38.8	190.7 ± 36.3	<0.001
Fasting blood glucose, mg/dL	98.0 ± 23.8	98.1 ± 26.5	97.5 ± 24.2	97.3 ± 22.0	99.2 ± 22.0	0.450
Hemoglobin A1c, %	5.721 ± 0.89	5.758 ± 0.99	5.741 ± 0.91	5.713 ± 0.87	5.671 ± 0.75	0.279

Values are shown as mean ± standard deviation or number (%). BMI, body mass index; AST, aspartate aminotransferase; ALT, alanine aminotransferase. P-for-trend values across quartiles of total abdominal muscle area for continuous variables were calculated using the Jonckheere–Terpstra test and were as follows: Age < 0.001, BMI < 0.001, Hemoglobin < 0.001, Albumin = 0.006, ALT < 0.001, Total cholesterol < 0.001, White blood cell = 0.383, Platelet = 0.750, Total bilirubin < 0.001, AST = 0.006, Fasting blood glucose = 0.450, Hemoglobin A1c = 0.279.

**FIGURE 2:**
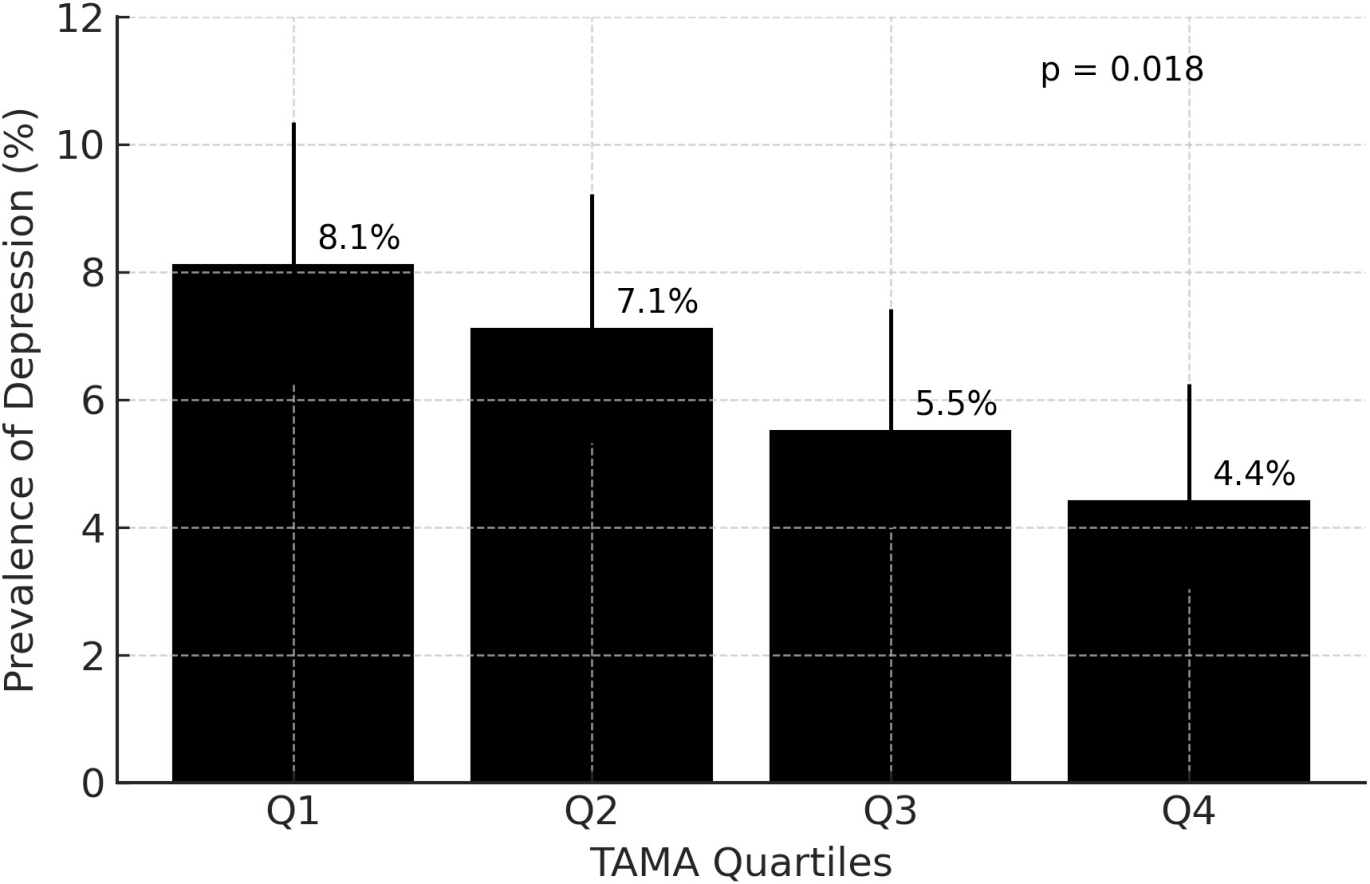
Prevalence of depression across total abdominal muscle area quartiles. Q, quartile; TAMA, total abdominal muscle area.

Figure 2 shows the prevalence of depression across TAMA quartiles. Depression was most prevalent in the lowest quartile (Q1, 8.1% [*n* = 59]) and exhibited a progressive decline with increasing TAMA, reaching 4.4% in the highest quartile (Q4, [*n* = 30]). The observed decreasing trend in depression prevalence across the TAMA quartiles was statistically significant (*p* = 0.018).

Table 2 presents the relationship between TAMA and depression, as determined through multivariable logistic regression analysis. In the unadjusted model (Model 1), individuals in the fourth quartile (Q4) exhibited significantly lower odds of depression compared to those in the first quartile (Q1) (OR: 0.515; 95% CI: 0.328–0.810; *p* = 0.004). This association remained statistically significant after adjusting for age and BMI (Model 2: OR: 0.519; 95% CI: 0.293–0.919; *p* = 0.024) and was consistent in Model 3, which further adjusted for lifestyle and clinical factors. In the fully adjusted model (Model 4), which incorporated biochemical markers, participants in Q4 had a 46.6% lower odds of depression compared to those in Q1 (OR: 0.534; 95% CI: 0.297–0.958; *p* = 0.035). Although the intermediate quartiles demonstrated a trend toward reduced odds of depression, these associations did not achieve statistical significance across all models.

**TABLE 2:** Association between total abdominal muscle area and depression according to quartiles of total abdominal muscle area.

Quartile of total abdominal muscle area	Odds ratio (95% CI)	*P*-value
**Model 1**		
Quartile 1 (reference)	1	
Quartile 2	0.864 (0.585–1.276)	0.464
Quartile 3	0.662 (0.438–1.000)	0.050
Quartile 4	0.515 (0.328–0.810)	0.004
**Model 2**		
Quartile 1 (reference)	1	
Quartile 2	0.864 (0.571–1.308)	0.491
Quartile 3	0.666 (0.417–1.064)	0.089
Quartile 4	0.519 (0.293–0.919)	0.024
**Model 3**		
Quartile 1 (reference)	1	
Quartile 2	0.923 (0.608–1.402)	0.709
Quartile 3	0.704 (0.439–1.130)	0.146
Quartile 4	0.553 (0.309–0.988)	0.045
**Model 4**		
Quartile 1 (reference)	1	
Quartile 2	0.912 (0.597–1.393)	0.671
Quartile 3	0.660 (0.407–1.070)	0.092
Quartile 4	0.534 (0.297–0.958)	0.035

CI, confidence interval. Model 1: Unadjusted. Model 2: Adjusted for age, body mass index. Model 3: Model 2 + smoking status, alcohol use, physical activity status, hypertension, and diabetes. Model 4: Model 3 + hemoglobin, albumin, total bilirubin, aspartate aminotransferase, alanine aminotransferase, total cholesterol, and hemoglobin A1c. P-for-trend was calculated using the median value of each quartile as a continuous variable in logistic regression: Model 1 < 0.001, Model 2 = 0.002, Model 3 = 0.015, Model 4 = 0.023.

## Discussion

This study investigated the relationship between abdominal muscle mass, as measured by CT, and the prevalence of depression among middle-aged and older Korean men. Our findings indicate that a higher TAMA is significantly associated with a reduced prevalence of depression. This inverse relationship remained significant even after adjusting for potential confounding factors, including demographic, lifestyle, clinical, and biochemical variables. These results suggest that abdominal muscle mass may be a marker of mental health in aging male populations.

The inverse association between abdominal muscle mass and depression identified in this study aligns with previous research highlighting a link between low skeletal muscle mass and increased risk of depressive symptoms, particularly in aging populations (4, 20). However, most prior studies have focused on appendicular or whole-body muscle measurements assessed through DXA or BIA (5–7), which may not fully capture the regional muscle distribution relevant to physical and metabolic function. In contrast, this study employed CT to quantify abdominal muscle mass, providing a more precise and anatomically detailed assessment. Abdominal muscles are integral to postural support, mobility, and core stability (21, 22), as well as metabolic regulation (23), all of which have been implicated in the pathophysiology of depression (2). Therefore, abdominal muscle mass measured by CT may serve as a functionally meaningful indicator that reflects both physical health and mental well-being.

Several biological and behavioral mechanisms may explain the inverse relationship between abdominal muscle mass and depression observed in this study. Skeletal muscle serves not only as a structural component but also as an endocrine organ, secreting myokines–bioactive peptides such as brain-derived neurotrophic factor, interleukin-6, and irisin–that play pivotal roles in neurogenesis, synaptic plasticity, immune modulation, and the regulation of mood and cognition (24, 25). A reduction in muscle mass may result in decreased myokine production, potentially impairing central nervous system function and contributing to depressive symptoms (26). Additionally, low muscle mass is frequently associated with increased visceral adiposity, systemic inflammation, and insulin resistance (8), all of which have been implicated in the pathophysiology of depression through inflammatory cytokine release and hypothalamic-pituitary-adrenal (HPA) axis dysregulation (27, 28). Higher total abdominal muscle area was associated with a greater proportion of alcohol users, which corresponded with slightly higher AST and ALT levels across quartiles; however, these enzyme levels remained within normal limits, suggesting limited clinical significance. Individuals with greater muscle mass may also engage in higher levels of physical activity and maintain functional independence (29), which are behavioral factors known to enhance mood, reduce stress, and improve psychological resilience, thereby further mitigating the risk of depression.

Our findings indicate that increased TAMA correlates with more advantageous metabolic and nutritional profiles, characterized by elevated levels of hemoglobin, albumin, and total bilirubin. These biomarkers may reflect overall health status and could be associated with the link between muscle mass and depression. Therefore, the fully adjusted model should be interpreted as a health-status–adjusted model rather than as a causal mediation model. For instance, hypoalbuminemia has been associated with chronic inflammation, frailty, and poor nutritional status, which are linked to an increased susceptibility to depressive symptoms (30). Similarly, anemia and low hemoglobin levels may lead to fatigue, reduced physical capacity, and decreased cerebral oxygenation, contributing to both cognitive decline and mood disorders (31). Although total bilirubin is often considered in the context of liver function, it also possesses antioxidant properties that may help mitigate oxidative stress, a process increasingly recognized in the pathogenesis of depression (32). Consequently, abdominal muscle mass, as quantified by CT, may reflect not only musculoskeletal integrity but also an individual’s broader physiological reserve and resilience against mood disturbances.

We identified a distinct inverse relationship between TAMA and the prevalence of depression, characterized by a progressive decrease in the proportion of individuals experiencing depressive symptoms across TAMA quartiles. This association remained robust after adjusting for a comprehensive array of potential confounders, including demographic, lifestyle, clinical, and biochemical variables. Notably, only participants in the highest TAMA quartile exhibited statistically significant reductions in depression risk across all multivariate models, while those in the second and third quartiles showed non-significant or borderline associations. This pattern may indicate a threshold effect, suggesting that only individuals with relatively high levels of abdominal muscle mass experience substantial mental health benefits. In other words, modest increases in muscle mass may not suffice to influence mood-related outcomes unless a critical volume or quality is attained. This observation highlights the importance of identifying clinically meaningful cut-off points and suggests that future research should also consider muscle quality such as density or lipid infiltration, which may provide further insights into the relationship between muscle composition and psychological well-being.

Several limitations must be considered when interpreting the findings of this study. Firstly, the cross-sectional design precludes any conclusions regarding the causal relationship between abdominal muscle mass and the prevalence of depression. Secondly, the study cohort consisted exclusively of Korean men undergoing routine health screening at a single tertiary medical center, which may introduce selection bias. Participants who voluntarily receive abdominopelvic CT tend to represent a subgroup with greater health awareness, distinctive lifestyle characteristics, or higher socioeconomic status, potentially limiting the generalizability of the findings. Therefore, the results may not be directly applicable to women, individuals of other ethnic backgrounds, or populations with different sociodemographic or clinical characteristics, and validation in more diverse cohorts is warranted. Thirdly, depression was assessed using the BDI, a widely validated but self-reported measure that may be prone to recall and social desirability biases. Furthermore, information on psychiatric history and antidepressant use was not available, and thus alternative diagnostic thresholds could not be meaningfully validated. Although a validated BDI cut-off (≥16) was applied, some degree of depression misclassification cannot be excluded. In addition, abdominal muscle quantification was performed exclusively using contrast-enhanced CT scans obtained during the portal venous phase. Although a standardized acquisition protocol was applied, contrast administration may influence Hounsfield unit HU attenuation and segmentation thresholds. Because non-contrast scans were not available for comparison, sensitivity analyses based on contrast status could not be performed, and this may introduce minor measurement variability. Lastly, lifestyle factors such as alcohol consumption and physical activity were assessed using self-reported questionnaires, which may lead to misclassification, and possible interactions between these behaviors and abdominal muscle mass could not be fully evaluated. Although we adjusted for a comprehensive range of demographic, lifestyle, clinical, and biochemical variables, residual confounding cannot be excluded, particularly from unmeasured psychosocial factors such as perceived stress, socioeconomic status, and sleep quality, which are known to influence the risk of depression.

In conclusion, higher abdominal muscle mass, as measured by CT, was independently associated with a lower prevalence of depression in Korean men aged 40 years and older. These findings suggest the potential significance of maintaining muscle mass for mental health in aging populations. Longitudinal studies are necessary to investigate the causal direction of this association and to assess whether interventions aimed at enhancing or preserving abdominal muscle mass may aid in the prevention of depression.

## Data Availability

The datasets used and/or analyzed in the current study are not publicly available due to institutional and privacy restrictions but are available from the corresponding author upon reasonable request. Requests to access the datasets should be directed to D-HL, dlaengh@hanmail.net.

## Supplementary material

Supplementary Figure 1 Segmental abdominal muscle analysis at the L3 vertebra on abdominopelvic computed tomography. TAMA, total abdominal muscle area including psoas, paraspinal, transversus abdominis, rectus abdominis, quadratus lumborum, and internal/external oblique muscles; LAMA, low-attenuation abdominal muscle area (−29 to 29 HU), indicative of low-density muscle and potential intramuscular fat; NAMA, normal-attenuation abdominal muscle area (30–150 HU); EMCLA, extramyocellular lipid area (−190 to −30 HU), representing lipid infiltration within the muscle compartment; HU, Hounsfield units.
