# Supplementary material for: Association between abdominal muscle mass quantified by computed tomography and depression in middle-aged and older Korean men: a cross-sectional study of 2,877 cases
Source: Front Med (Lausanne). 2026 Mar 12;13:1656330. doi: 10.3389/fmed.2026.1656330 (PMC13017955; doi:10.3389/fmed.2026.1656330)
Supplement: Supplementary file 1 [file Table_1.DOCX]

**Supplementary table 1.** Variance inflation factor (VIF) for abdominal muscle mass (TAMA) and body mass index (BMI) in multivariable logistic regression

| **Variable** | **B** | **SE** | **beta** | **t** | **p-value** | **Tolerance** | **VIF** |
| --- | --- | --- | --- | --- | --- | --- | --- |
| **BMI (kg/m²)** | -0.002 | 0.002 | -0.020 | -0.828 | 0.408 | 0.593 | 1.687 |
| **Total abdominal muscle area (cm²)** | 0.000 | 0.000 | -0.041 | -1.684 | 0.092 | 0.593 | 1.687 |

VIF < 5 was considered indicative of no significant multicollinearity.

**Supplementary table 2.** Model Diagnostics for Logistic Regression Evaluating the Association Between Total Abdominal Muscle Area (TAMA) and Depression

| **Diagnostic Method** | **Model 1** | **Model 2** | **Model 3** | **Model 4** |
| --- | --- | --- | --- | --- |
| **Calibration (Hosmer–Lemeshow Test)** | χ² = 6.21, p = 0.401 | χ² = 7.03, p = 0.318 | χ² = 8.17, p = 0.238 | χ² = 9.51, p = 0.215 |
| **Discrimination (AUC)** | 0.61 (95% CI: 0.57–0.65) | 0.64 (95% CI: 0.60–0.68) | 0.67 (95% CI: 0.63–0.71) | 0.70 (95% CI: 0.66–0.74) |
| **Pseudo-R² (Nagelkerke)** | 0.021 | 0.048 | 0.083 | 0.118 |
| **Linearity of Logit (Box–Tidwell Test*)** | Not applicable | Age (p = 0.241), BMI (p = 0.387) | All p > 0.10 | All p > 0.10 |
