# Supplementary figures and images for: Association between abdominal muscle mass quantified by computed tomography and depression in middle-aged and older Korean men: a cross-sectional study of 2,877 cases
Source: Front Med (Lausanne). 2026 Mar 12;13:1656330. doi: 10.3389/fmed.2026.1656330 (PMC13017955; doi:10.3389/fmed.2026.1656330)

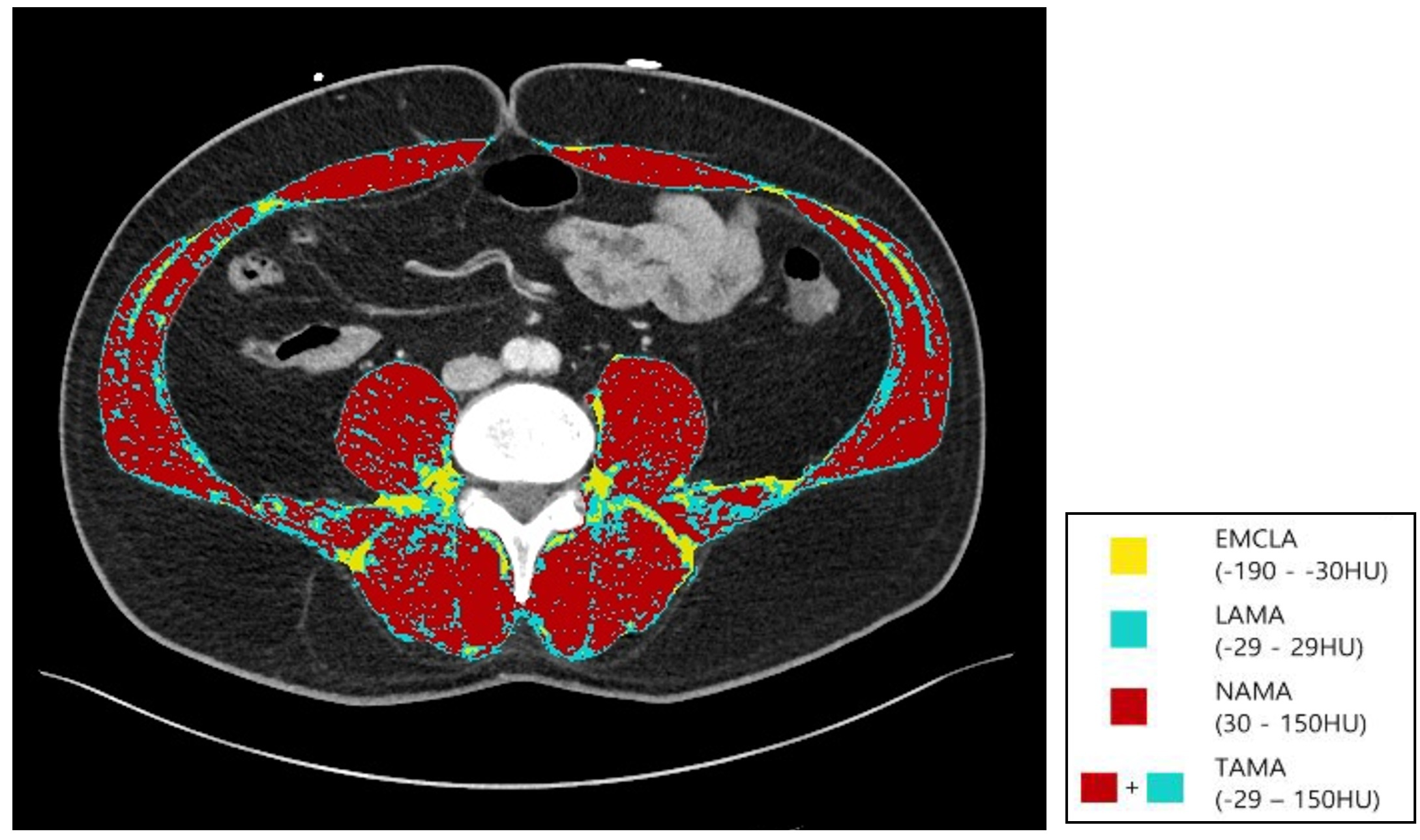

Supplement: Supplementary Figure 1 — Segmental abdominal muscle analysis at the L3 vertebra on abdominopelvic computed tomography. TAMA, total abdominal muscle area including psoas, paraspinal, transversus abdominis, rectus abdominis, quadratus lumborum, and internal/external oblique muscles; LAMA, low-attenuation abdominal muscle area (−29 to 29 HU), indicative of low-density muscle and potential intramuscular fat; NAMA, normal-attenuation abdominal muscle area (30–150 HU); EMCLA, extramyocellular lipid area (−190 to −30 HU), representing lipid infiltration within the muscle compartment; HU, Hounsfield units. [file Image_1.TIF]
